# Differences of the Structure of Immune Regulatory Cell Populations between Cellular Material from Sonographically Detected Focal Thyroid Lesions and Peripheral Blood in Humans

**DOI:** 10.3390/ijms20040918

**Published:** 2019-02-20

**Authors:** Mariusz Stasiołek, Przemysław W. Śliwka, Magdalena Stasiak, Kinga Krawczyk-Rusiecka, Elżbieta Skowrońska-Jóźwiak, Zbigniew Adamczewski, Andrzej Lewiński

**Affiliations:** 1 Department of Neurology, Medical University of Lodz, 90-153 Lodz, Poland; mariusz.stasiolek@umed.lodz.pl; 2 Department of Endocrinology and Metabolic Diseases, Polish Mother’s Memorial Hospital—Research Institute, 93-338 Lodz, Poland; p.sliwka87@gmail.com (P.W.Ś.); mstasiak33@gmail.com (M.S.); kinga.krawczyk-rusiecka@umed.lodz.pl (K.K.-R.); elzbieta.skowronska-jozwiak@umed.lodz.pl (E.S.-J.); zbadam@o2.pl (Z.A.); 3 Department of Endocrinology and Metabolic Diseases, Medical University of Lodz, 93-338 Lodz, Poland

**Keywords:** dendritic cells, monocytes, thyroid, thyroid focal lesion, autoimmune thyroid disease

## Abstract

Focal thyroid lesions are common ultrasound findings with the estimated prevalence up to 67% of the population. They form characteristically enveloped regions with individual encapsulated microenvironment that may involve the specific distribution of immune system compounds—especially antigen presenting cells (APC). We analyzed and compared the most potent APC—plasmacytoid and conventional dendritic cells (DCs) subpopulations and three monocyte subpopulations as well as other immune cells—in peripheral blood and local blood of thyroid gland obtained parallelly in patients with focal thyroid lesions using flow cytometry. The analysis revealed significant differences in the distribution of main subsets of assessed cells between peripheral blood and biopsy material. The results support the existence of local, organ-specific immune reaction control networks within thyroid nodules.

## 1. Introduction

Focal lesions of the thyroid gland are differentiated fragments of organ tissue characterized by disturbed structure of the parenchyma, visible in ultrasonography (US) as isolated areas of altered echogenicity. Palpable thyroid nodules occur in 5% of the population, however, on the basis of neck US it is suggested that thyroid nodules can be present in 20–70% of the total population [1,2]. Many thyroid pathological processes—such as nodular goiter, autoimmune disease, or cancer—are associated with focal lesions of the thyroid gland.

Thyroid nodules often represent characteristically enveloped regions with individual encapsulated microenvironment. The involvement of the immune system elements, present both in the tissue and in the local organ blood circulation, is postulated in the pathogenesis of both benign and malignant thyroid lesions. This applies in particular to antigen presenting cells (APC).

While most of focal thyroid lesions are of benign nature, thyroid carcinomas occur in approximately 4–5% of palpable nodules [1]. Thyroid focal lesions may develop in the course of malignant diseases of the gland (i.e., papillary, follicular, medullary, or anaplastic thyroid carcinoma) or in the case of lymphomas, as well as non-malignant tumors such as adenomas. Formation of thyroid nodule may also occur during different types of inflammatory thyroid disorders, i.e., Hashimoto thyroiditis (HT), Graves’ disease (GD), or subacute thyroiditis. Interestingly, significant differences in immune system components, including APC, can be found between the peripheral blood and the local blood flow in autoimmune thyroid diseases (AITD) as well as in other organ-specific pathological processes [3,4,5].

APC are heterogeneous group of cells that exhibit common ability to capture and process antigens in order to present them in the context of appropriate major histocompatibility complex (MHC) molecules [6]. Among different APC populations, dendritic cells (DCs) and monocytes were shown to poses particularly strong immunoregulatory properties and—accordingly—an engagement of these cells has been suggested in various pathological processes.

It is believed that DCs—the most effective type of APC—play a critical role in the control of the function of the immune system, also as a component of local thyroid regulatory systems. Healthy thyroid tissue is characterized by a small amount of DCs located outside thyroid follicles. Increased number of DCs infiltrating the thyroid gland was found in the course of AITD and thyroid cancer [4,7].

The immunoregulatory function differs considerably among specific DC subpopulations. Two main lineage backgrounds, which provide cells characterized by distinct functional properties, surface molecule expression, efficacy of antigen uptake and presentation, as well as cytokine secretion profile were described [8]. Conventional DCs (cDCs) subset, also known as myeloid DCs, is believed to be involved in typical T helper (Th)1 and/or Th17 immune response promotion. Conventional DCs secrete large amounts of IL-6 and TNF-α and are also considered as a source of large amounts of other pro-inflammatory cytokines such as IL-12 and IL-23 [9,10]. It is believed that this type of strong pro-inflammatory reaction in response to the components of pathogens may play a role in the induction of autoaggressive processes [11]. The other main DC subset—plasmacytoid DCs (pDCs)—is suggested as crucial for Th2 and regulatory T cells generation. Whereas, in the innate immune system, pDCs are known as one of the main sources of type I interferons (IFN I) [12,13].

Monocytes originate from bone marrow myeloid progenitor and represent 5–10% of human peripheral blood leukocyte population. After migration to target organs they differentiate into macrophages or DCs [14]. It is generally accepted that—although more numerous—monocytes are less potent APC than DCs and have lower ability to drive immune responses. The role of monocytes as elements of the innate immune response is not limited to antigen intake and presentation. Depending on multiple microenvironmental factors (e.g., pathogen-derived products) monocytes may be a potent source of a variety of proinflammatory and/or regulatory cytokines [15,16,17]. Moreover, direct interaction with effector cells exerts further differentiating effects on monocyte function [18,19].

Based on the surface expression of CD14 (monocyte specific lipopolysaccharide (LPS) receptor) and CD16 (Fc type G immunoglobulin receptor (FcγRIII)), human peripheral blood monocytes were divided into three functionally different subpopulations: CD14^high^CD16^−^ classical monocytes, CD14^high^CD16^+^ intermediate monocytes and CD14^low^CD16^+^ non-classical monocytes [20]. The CD16^+^ non-classical and intermediate monocyte subsets are generally termed proinflammatory monocytes because of their ability to secrete high amounts of TNF-α and IL-1β [21,22]. However, the exact role of particular CD16^+^ monocyte subsets in promotion, propagation and maintaining of immune reaction remains to be elucidated [23]. 

Presence of various populations of APC in the thyroid and their potential involvement in the pathomechanisms of different thyroid disorders have been indicated in earlier studies [4]. However, the knowledge about the role of DCs and other APC (e.g., monocyte subtypes) in the local thyroid regulatory mechanisms remains scarce and there is virtually no information about the nature of interaction between DCs, other immune cells and thyrocytes under physiological and pathological conditions. 

The aim of this study was to analyze and compare APC subpopulations in peripheral blood and local blood flow of thyroid gland in patients with focal thyroid lesions.

## 2. Results

Cytometric analysis of peripheral blood and fine needle aspiration biopsy (FNAB) samples showed differences in structure and phenotype of the studied populations of immune cells.

### 2.1. Monocytes

**Figure 1:**
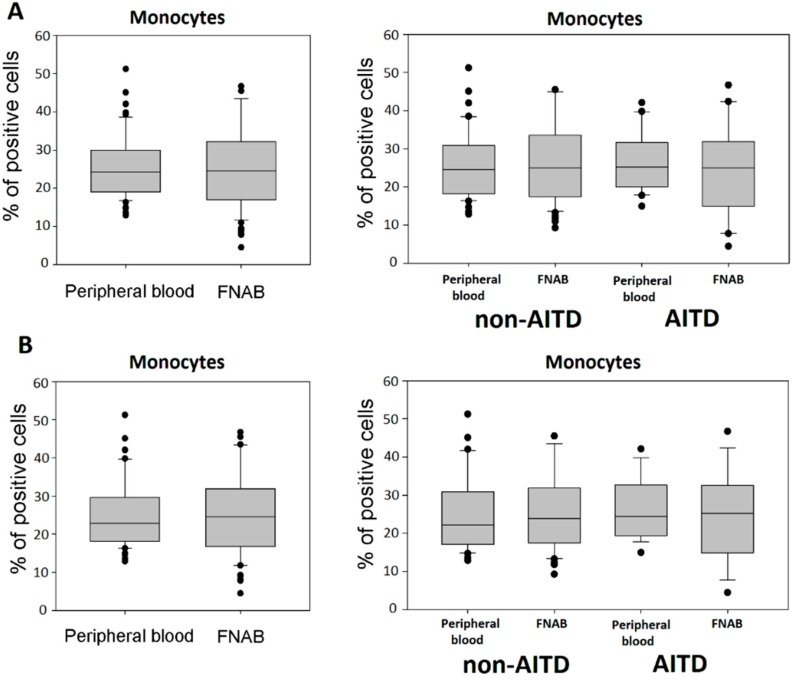
Percentage of monocytes in the leukocyte mononuclear fraction of peripheral blood and FNAB, respectively; in entire cohort and in particular patient groups; (**A**) all patients; (**B**) euthyroid patients.

The percentage of CD14+ monocytes in leukocyte mononuclear fraction did not differ between FNAB and peripheral blood. Similarly, no difference was observed in particular patient groups (Figure 1). In order to exclude the possible impact of differences in the thyrometabolic state on studied parameters, analogous analysis was performed in the group of exclusively euthyroid patients, which did not reveal any differences in the percentage of monocytes (Figure 1).

**Figure 2:**
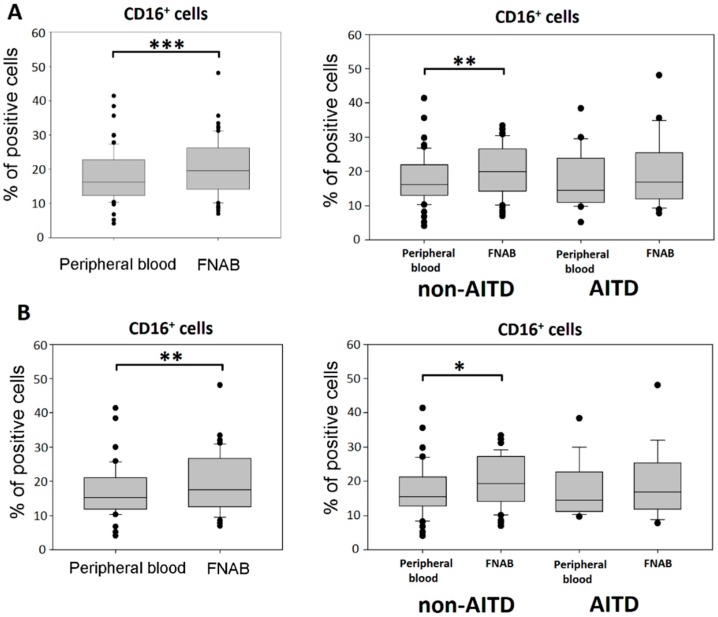
Percentage of CD16^+^ cells in the leukocyte mononuclear fraction of peripheral blood and FNAB, respectively; in entire cohort and in particular patient groups; (**A**) all patients; (**B**) euthyroid patients (* *p* < 0.05, ** *p* < 0.01, *** *p* < 0.001).

**Figure 3:**
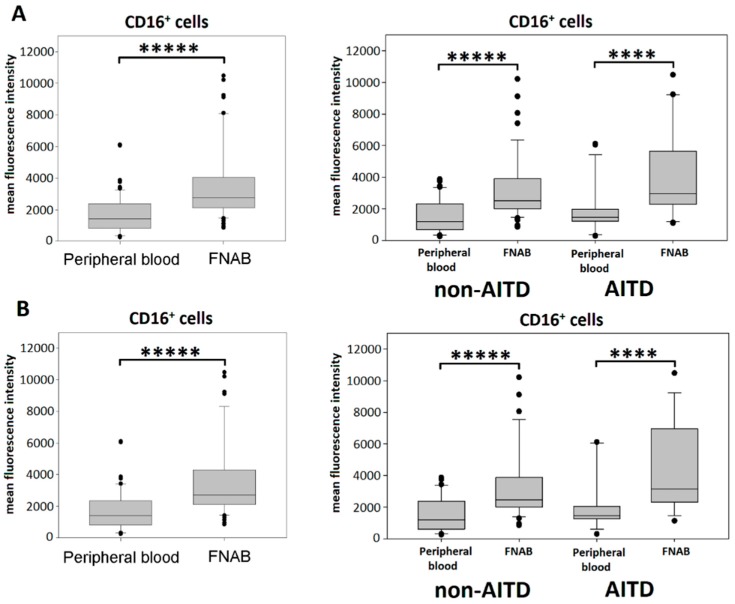
Mean fluorescence intensity for CD16 staining; in entire cohort and in particular patient groups of peripheral blood and FNAB, respectively; (**A**) all patients; (**B**) euthyroid patients (**** *p* < 0.0001, ***** *p* < 0.00001).

Significantly higher expression of CD16 molecule was shown in the leukocyte mononuclear fraction of FNAB as compared to peripheral blood (Figure 2). Interestingly, CD16^+^ cells from FNAB were characterized by significantly higher mean fluorescence of CD16 staining (*p* < 0.00001) indicating higher level of surface expression of this molecule (Figure 3). There was no difference in the mean fluorescence value of the CD16 staining between monocyte and lymphocyte fraction, neither in FNAB nor peripheral blood samples.

Similarly to the entire cohort, statistically significant higher percentages of CD16^+^ cells were found in FNAB material of euthyroid patients as compared to their peripheral blood (Figure 2).

Analysis in individual groups of patients showed the difference in the percentage of CD16^+^ cells between peripheral blood and FNAB material in the group of non-AITD patients only. Neither the percentage of CD16^+^ cells nor the average fluorescence value of this molecule differed statistically between the groups of patients (Figure 2).

**Figure 4:**
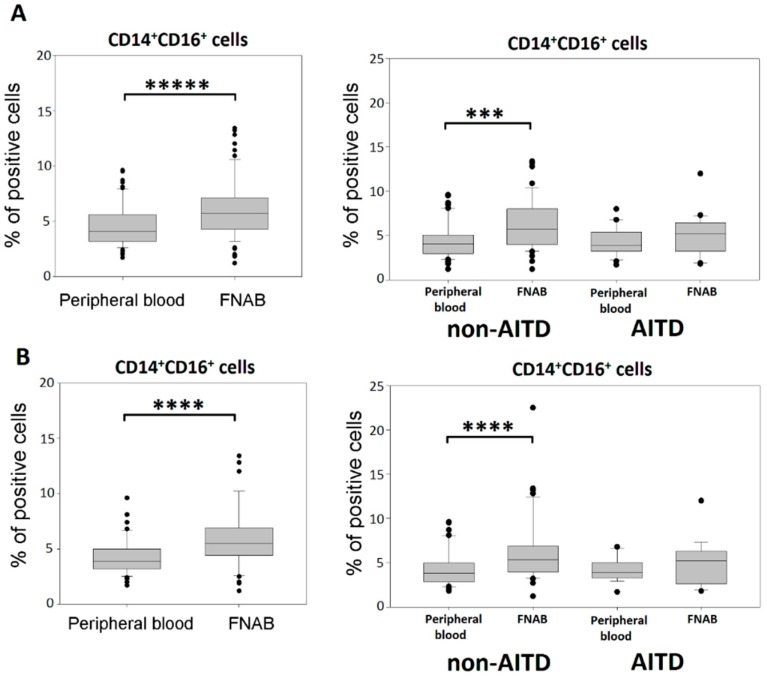
Percentage of CD14^+^CD16^+^ monocytes in the leukocyte mononuclear fraction of peripheral blood and FNAB, respectively; in entire cohort and in particular patient groups; (**A**) all patients; (**B**) euthyroid patients (*** *p* < 0.001, **** *p* < 0.0001, ***** *p* < 0.00001).

The CD16 molecule can be expressed both by monocytes and other cells e.g., NK cells. Significantly higher percentage of CD14^+^CD16^+^ monocytes was shown in FNAB samples in the whole cohort as wells as in non-AITD patients. These findings were independent of thyrometabolic state (Figure 4). Whereas, there was no difference between the FNAB and peripheral blood cells in the percentage of CD14^−^CD16^+^ non-monocytes. 

### 2.2. Monocyte Subsets

**Figure 5:**
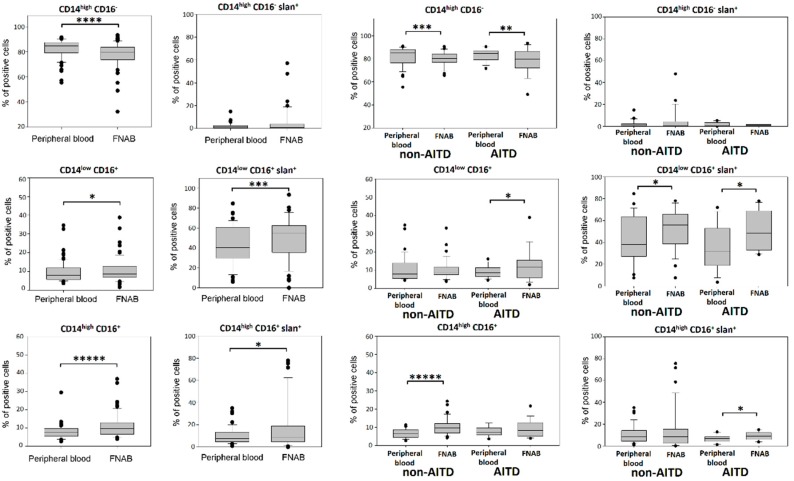
Percentage of monocyte subsets in the leukocyte mononuclear fraction of peripheral blood and FNAB, respectively; in entire cohort and in particular patient groups (* *p* < 0.05, ** *p* < 0.01, *** *p* < 0.001, **** *p* < 0.0001, ***** *p* < 0.00001).

**Figure 6:**
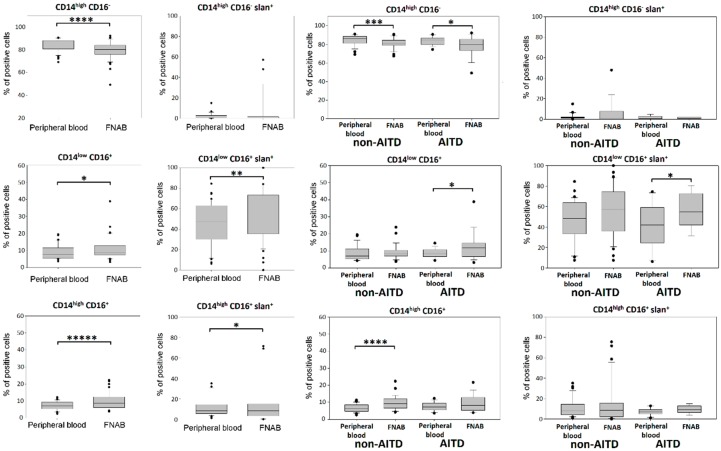
Euthyroid patients. Percentage of monocyte subsets in the leukocyte mononuclear fraction of peripheral blood and FNABs, respectively; in entire cohort and in particular patient groups (* *p* < 0.05, ** *p* < 0.01, *** *p* < 0.001, **** *p* < 0.0001, ***** *p* < 0.00001).

In further steps, we performed the analysis of the structure of monocyte subsets characterized on the basis of CD14 and CD16 expression. Significantly lower percentage of classical monocytes was observed in the FNAB material than in the peripheral blood. In contrast, the percentages of monocytes of the intermediate and non-classical subpopulations were significantly higher in the FNAB (Figure 5). These findings were independent of thyrometabolic state of patients (Figure 6).

The next parameter analyzed was the expression of the slan molecule on the surface of monocytes. Slan (6-Sulfo LacNAc—a carbohydrate modification of P-selectin glycoprotein ligand-1 (PSGL-1)) was described as a marker of new subset of DC/monocyte [23]. In agreement with earlier publications—in our experimental setting CD14^low^CD16^+^ monocyte subset showed the highest percentage of slan expression, both in peripheral blood and in FNAB samples. Moreover, this parameter reached significantly higher value for CD14^low^CD16^+^ and CD14^high^CD16^+^ monocyte subsets in FNAB as compared to peripheral blood and these results were independent of thyrometabolic state of patients (Figure 5 and Figure 6).

There were no differences in the monocyte population structure between patient groups. However, in both non-AITD and AITD group statistically lower percentage of classical CD14^high^CD16^−^ monocytes was shown in FNAB material as compared to peripheral blood. Interestingly, the associated shift of monocyte structure in FNAB material was expressed differently in particular patient groups. Higher percentage of CD14^high^CD16^+^ intermediate monocytes was shown in FNABs of non-AITD patients while AITD patients had higher percentage of CD14^low^CD16^+^ non-classical monocytes (Figure 5). These findings were independent of thyrometabolic state (Figure 6). 

Analysis of the percentage of slan^+^ cells did not show any significant differences between patient groups. In non-AITD patient group the percentage of CD14^low^CD16^+^slan^+^ non-classical monocytes was higher in FNAB material. However, this difference was not shown when only euthyroid patients were included in the analysis. In AITD patient group, percentages of CD14^high^CD16^+^slan^+^ and CD14^low^CD16^+^slan^+^ monocytes were higher in FNAB material. However, in the euthyroid patients the statistically significant difference remained in the case of CD14^low^CD16^+^slan^+^ monocytes only (Figure 5).

### 2.3. Conventional DCs

**Figure 7:**
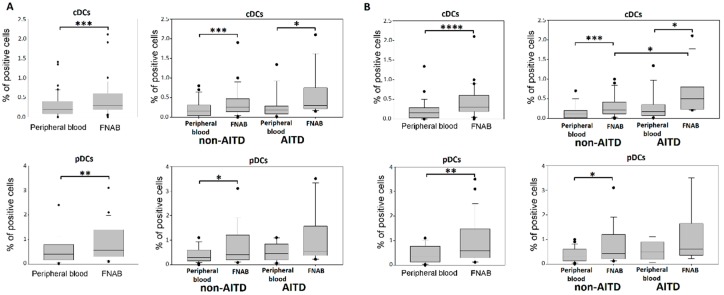
Percentage of DCs in the leukocyte mononuclear fraction of peripheral blood and FNAB, respectively in entire cohort and in particular patient groups: (**A**) all patients; (**B**) euthyroid patients (* *p* < 0.05, ** *p* < 0.01, *** *p* < 0.001, **** *p* < 0.0001).

Significantly higher percentage of cDCs in the FNAB material was observed as compared to peripheral blood. The difference remained statistically significant in both patient groups. The higher percentage of cDCs in the FNAB material was also shown in the analysis performed in euthyroid patients. Additionally, in euthyroid patients, the percentage of cDCs in FNAB was higher in AITD than in non-AITD group (Figure 7).

### 2.4. Plasmacytoid DCs

The percentage of pDCs was significantly higher in the FNAB material than in the peripheral blood. Interestingly, in the analysis of particular patient groups this difference remained significant in non-AITD group only. The higher percentage of pDCs in the FNAB material was confirmed in euthyroid patients. There were no significant differences in the percentage of pDCs between particular patient groups (Figure 7).

### 2.5. NK Cells

**Figure 8:**
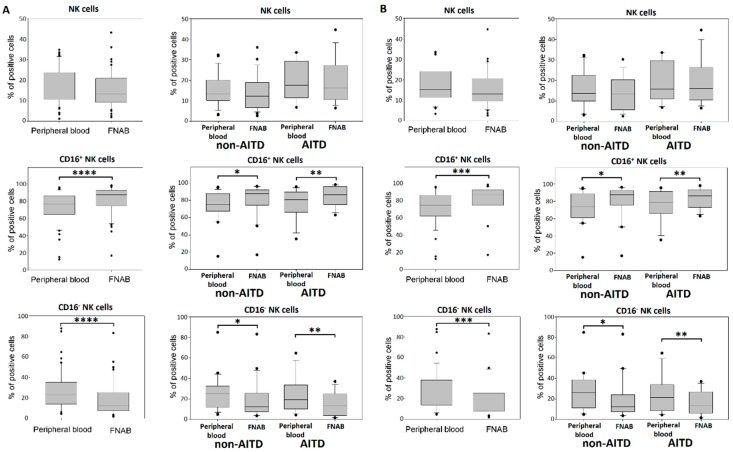
Percentage of NK cells and their subtypes in the lymphocyte fraction of peripheral blood and FNABs, respectively. in entire cohort and in particular patient groups: (**A**) all patients; (**B**) euthyroid patients (* *p* < 0.05, ** *p* < 0.01, *** *p* < 0.001, **** *p* < 0.0001).

The analysis revealed no difference in the percentage of NK cells in the lymphocyte fraction between the FNAB material and peripheral blood. However, statistically significant differences were found in the NK cells subtypes. The lymphocyte fraction of the FNAB material was characterized by a significantly higher percentage of CD3^−^CD56^+^CD16^+^ NK cells and a lower percentage of CD3^−^CD56^+^CD16^−^NK cells as compared to peripheral blood (Figure 8). Similar differences in the structure of CD16^+^ and CD16^−^ NK cell subtypes between the FNAB material and peripheral blood were observed in both patient groups. Whereas, the percentage of NK cell subsets did not differ between particular patient groups. The findings were independent of thyrometabolic state (Figure 8).

### 2.6. NKT Cells

**Figure 9:**
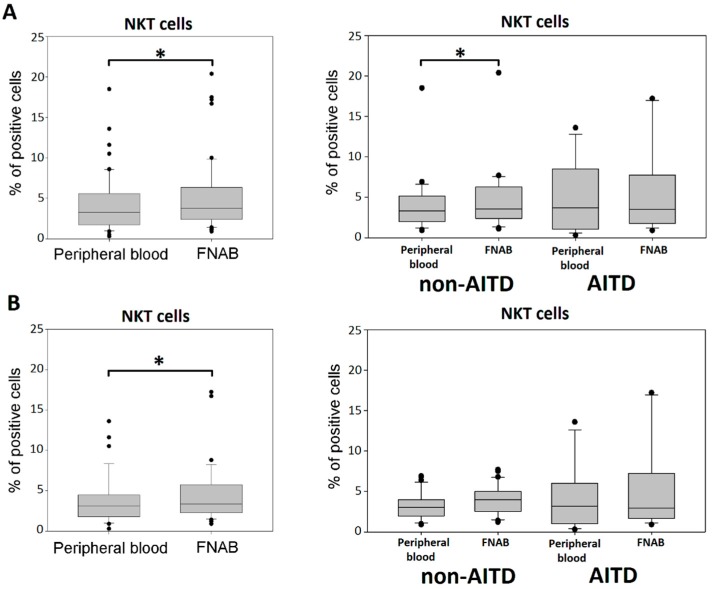
Percentage of NKT cells in the lymphocyte fraction of peripheral blood and FNABs, respectively in entire cohort and in particular patient groups; (**A**) all patients; (**B**) euthyroid patients (* *p* < 0.05).

Significantly higher percentage of NKT cells was shown in the lymphocyte fraction of FNAB material than in peripheral blood. The difference was also observed in euthyroid patients only. There were no differences between patient groups. However, the analysis within particular patient groups revealed significant difference between the FNAB material and peripheral blood only for the whole non-AITD group. There were no differences between patient groups (Figure 9).

## 3. Discussion

Focal lesions of thyroid gland represent a micro-environment in which many processes, both at the cellular and subcellular levels, may be different than in the case of healthy glandular tissue. Accumulating body of evidence, supported also by our earlier publications, suggests the existence of local tissue-specific immunoregulatory systems characterized, e.g., by different structure and function of APC subpopulations [5,24]. Most importantly, disturbances of the local immune environment may potentially play an important role in organ-specific pathological processes. Meanwhile, this issue is rather poorly characterized in the case of the thyroid gland and its diseases, particularly associated with formation of focal lesions. 

In our study we report, for the first time, significant differences in the distribution of main monocyte subsets between peripheral blood and local thyroid blood in patients with thyroid nodules. Leukocyte fraction of FNAB specimens was characterized by increased percentage of CD14^high^CD16^+^ and CD14^low^CD16^+^ monocyte subsets and decreased percentage of CD14^high^CD16^−^ classical monocytes. Interestingly, the above-mentioned parameters differed between AITD and non-AITD patient groups. These observations suggest different involvement of individual monocyte subsets in local regulatory processes in thyroid diseases of various background (e.g., autoimmune vs. non-autoimmune). The important role of CD14^high^CD16^+^ monocytes is suggested in many inflammatory conditions [23]. An increased percentage of this monocyte population was found in the peripheral blood of patients with sepsis, in the cases of tuberculosis, or in primary idiopathic peripheral vasculitis (Eales disease) [23] as well as peripheral arterial disease [25]. Most relevant seem to be the results of studies performed on target organ tissue in inflammatory conditions. The CD14^high^CD16^+^ subpopulation represented a large percentage of monocytes in inflammatory infiltrates of the intestinal mucosa in Crohn’s disease [26], as well as among inflammatory cells of the synovial fluid in patients with rheumatoid arthritis [27]. In our study higher percentage of CD14^high^CD16^+^ monocyte subpopulation was found in non-AITD group, while FNAB material of AITD patients showed an increased percentage of CD14^low^CD16^+^ but not CD14^high^CD16^+^ monocyte subset. Non-classical CD14^low^CD16^+^ cells are considered to be a more mature monocyte population [28], characterized by higher TNF-α production and a higher ability to stimulate autologous T lymphocytes to secrete pro-inflammatory cytokines [29]. Moreover, the high expression of cytoskeletal genes involved in cell mobility of non-classical monocytes grant them the ability to constant survey of the endothelium for signs of inflammation or damage [30,31]. Thus, higher percentage of non-classical monocytes in FNAB observed in our study may be explained by transmigration of CD14^low^CD16^+^ monocytes into the site of ongoing autoimmune reaction. It can, therefore, be assumed that depending on the type and severity of the immune response, monocytes of the intermediate and non-classical subsets may, in addition to increased presence in the systemic and local circulation, show an increased affinity to peripheral tissues in organ specific pattern.

In the course of further phenotype analysis of monocytes, it was found that the CD14^low^CD16^+^ and CD14^high^CD16^+^ monocyte subsets obtained from thyroid focal lesions were characterized by an increased percentage of cells with surface expression of the slan molecule in comparison with peripheral blood. The exact role and linear background of slan^+^ cells remain unclear. Slan^+^ cells were suggested to represent a distinct subtype of DCs—so called slanDCs [32]. SlanDCs express CD16 and low amount of CD14 which implies significant similarities with the monocyte subset—non-classical CD14^low^CD16^+^ monocytes. However, it was shown that the slan molecule is present on the surface of about 50% CD14^low^CD16^+^ monocytes only, therefore it is uncertain whether the slanDCs and CD14^low^CD16^+^ monocytes should be classified as the same population [23]. Nevertheless, cells expressing slan molecule are likely to be more proinflammatory and more potent APC [33]. As previously reported in the literature [30,34], also in the current analysis, a significant percentage of CD14^low^CD16^+^ monocytes expressed slan in both peripheral blood and FNAB material. Moreover, the difference in slan expression was dependent on the thyrometabolic state of patients. In euthyroid patients group only CD14^low^CD16^+^slan^+^ monocytes in FNAB material of AITD patients were significantly increased as compared to peripheral blood. These findings once again point to a local organ specific to residing monocyte subpopulations. 

A very interesting observation made in the present study is also the significantly higher level of CD16 surface expression on cells obtained from the thyroid gland. This observation was made in both patient groups and was independent of thyrometabolic state. The CD16 molecule binds IgG immune complexes and activates intracellular pathways involved in stimulation of the mechanisms of natural cytotoxicity, secretion of proinflammatory cytokines, as well as phagocytosis [35]. The CD16 upregulation on monocytes in local thyroid blood could be associated with the presence of transcription growth factor-β (TGF-β) in the nodular microenvironment. TGF-β is known to induce CD16 expression in monocytes [36] and was found to be upregulated in benign and malignant thyroid nodules compared to unaffected normal thyroid tissue [37]. On the other hand, thyrocytes are known to produce granulocyte/macrophage colony-stimulation factor (GM-CSF), which was found to antagonize the TGF-β induced expression of FcyRIII on human monocytes [38,39]. Deregulation of Fcγ receptors expression was found in the course of few human diseases, however, any functional relevance remains to be determined [40,41]. Increased expression of the CD16 molecule on the surface of leukocytes was reported in several inflammatory diseases. In comparison to healthy subjects, the expression of CD16 on the surface of peripheral blood mononuclear cells (PBMCs) was significantly higher in patients with HT [42]. Similar observation was also made in monocytes of patients with sarcoidosis [43] and rheumatoid arthritis [44] and patients with malaria [41]. In the present study, the CD16 mean fluorescence value did not differ significantly between particular groups of patients. There was no difference between the monocyte and the lymphocyte fraction. However, in order to get reliable conclusions studies in large, homogenous groups of patients with specific forms of AITD are needed, with focus laid on the CD16 expression in correlation with other Fc receptors.

The excessive engagement of DCs has been already suggested in the pathophysiology of different thyroid disorders as reviewed extensively in our previous publication [4]. Until recently, however, there was no information on the correlation between thyroid infiltrating DCs and DCs present in the peripheral blood of patients with thyroid diseases. Such analysis of DCs subsets simultaneously in the thyroid gland and in peripheral blood was performed for the first time by Leskela et al. [24] in patients with different forms of AITD. In both HT and GD patients the population of peripheral blood pDCs was significantly lower as compared to healthy controls, but no such difference was found for the cDCs population. It should be underlined, that in the group of AITD patients, with either GD or HT, the percentage of pDCs found in the thyroid tissue was significantly higher than that found in peripheral blood. However, in the above-mentioned study, the evaluation of immune cells in the thyroid was based on immunohistochemistry of gland fragments obtained during surgery. As it was indicated earlier, in the present work, the analysis was performed on the leukocyte fraction of blood cells collected during biopsy of the thyroid focal lesions, and the analysis in both peripheral blood and FNAB material was carried out under the same conditions using flow cytometry. Despite the methodological differences, our observations were similar to those of Leskela et al., as in the present study the pDC subset constituted higher percentage of the mononuclear fraction of leukocytes in the FNAB material as compared to the peripheral blood. In our current analysis, no differences in peripheral blood pDCs percentage were observed between patients with and without AITD. It should be emphasized, however, that unlike in the Leskela’s work, the structure of the current study was based on the analysis of paired FNAB and peripheral blood samples and did not assume a comparative analysis in a control group of healthy people.

The increased percentage of pDCs subpopulations in the FNAB mononuclear leukocyte fraction may be related to the response of these cells to chemotactic stimuli generated during a local inflammatory process occurring inside the focal lesions of the thyroid or in surrounding tissue. Plasmacytoid DCs are considered to be the main source of type I IFN secreted in the early response to pathogenic antigens. On the other hand, these cells are characterized by a strong ability to induce tolerance in the adaptive arm of the immune response, in the mechanisms dependent on deletion of CD8^+^ lymphocytes, anergy of CD4^+^ lymphocytes, or differentiation of regulatory T cells [45]. In normal circumstances, these processes play a key role in targeting, and control of the severity and duration of the immune response. Interestingly, these phenomena are also postulated as a component of tumor immune escape where tumor-infiltrating pDCs often tend to be tolerogenic [46]. Tolerogenic capacity of pDCs, however, strongly depends on the type of disease process in which these cells are involved. In the study by Mao et al. performed in GD patients peripheral blood pDCs were shown to have a strong ability to suppress regulatory T cell function in vitro and consequently promote proliferation of effector cells. Importantly, such a strong regulatory T cell inhibition was not observed in any of the cDCs or pDCs populations derived from euthyroid patients [47]. 

In addition to differences in pDCs subset, the current study showed an increase in percentage of cDCs in the FNAB material as compared to peripheral blood. Similarly, as in the case of pDCs, the increased presence of cDCs in the FNAB material can potentially be understood as a migration step from the systemic circulation to peripheral tissues. Whereas, the differences found between patients with different pathologies of the thyroid may be associated with different intensity of local inflammatory processes and intensity of chemotactic signals. The important thing is that DCs accumulating in target tissues, become themselves a very efficient source of a wide range of humoral factors, including IL 8—one of the key chemoattractants for granulocytes, monocytes, and lymphocytes [48]. Thus, a high percentage of many leukocyte populations in the thyroid FNAB material may be a secondary phenomenon to the influx of cells with strong immunoregulatory properties, such as DCs. 

In our study, we also demonstrated higher percentage of CD16^+^ NK cells in FNAB material. CD56^low^CD16^+^ NK cells constitute ca. 90% of NK cells in the bloodstream and are considered a highly cytotoxic subpopulation. In contrast, CD56^low^CD16^−^ NK cells are observed in the parenchyma of most organs. It is suggested that a high percentage of CD56^low^CD16^+^ NK cells in tissues may be associated with an intense cytotoxic reaction, e.g., against tumor cells or cells infected with intracellular pathogen. It may also indicate ongoing autoimmune response [49]. Importantly, the domination of CD16^+^ NK cells over CD16^−^ NK cells was also reported in the tissue of patients with non-toxic thyroid goiter [50]. Whereas, both subpopulations of NK cells were shown to be much more abundant in the papillary thyroid carcinoma than in the case of patients with non-toxic thyroid goiter [50,51].

In conclusion, in our work we demonstrated a broad set of data increasing the knowledge of local immune environment in focal thyroid lesions in humans. 

## 4. Materials and Methods

### 4.1. Patients

A group of 90 patients with thyroid focal lesions found on US examination were included into the study. Patients were diagnosed and recruited in the Department of Endocrinology and Metabolic Diseases—Polish Mother’s Memorial Hospital—Research Institute, Lodz, Poland. The study was conducted in accordance with the Declaration of Helsinki, and the protocol was approved by the Ethics Committee of the Polish Mother’s Memorial Hospital—Research Institute, Lodz, Poland (44/2014; 64/2015). Prior to the enrolment, all of the participants signed an informed consent, according to the study protocol. Analysis was performed on paired FNAB and peripheral blood samples obtained at the same time point. Complete clinical and demographic data were registered (Table 1).

On the basis of clinical diagnosis patients were divided into two groups: non-AITD patients (*n* = 67) and patients with AITD (*n* = 23). All analyses were performed additionally on analogous groups included only euthyroid patients (*n* = 72): non-AITD euthyroid patients (*n* = 51) and AITD euthyroid patients (*n* = 21). 

### 4.2. Ultrasound Guided FNAB Procedure and Peripheral Blood Collection

In every patient, US examination was performed in a supine position, using a Toshiba Aplio XG scanner (Toshiba, Japan), with high frequency 7–14 MHz linear transducer, with standard settings optimized for thyroid imaging. Fine needle aspiration biopsy was performed in all patients using a 23-gauge needle. All FNAB specimens were obtained from standard clinical indications by trained medical personnel under the control of US in the Department of Endocrinology and Metabolic Diseases—Polish Mother’s Memorial Hospital, Lodz, Poland. The FNAB needle after aspirate collection was immediately washed with phosphate buffered saline (PBS) (Hirszfeld Institute of Immunology and Experimental Therapy, Polish Academy of Sciences, Wroclaw, Poland) into EDTA containing Blood Collecting System (Sarstedt, Germany). Directly after FNAB procedure a sample of peripheral blood (2.7 mL) was collected from each patient by venipuncture into EDTA containing Blood Collecting System (the same type as in the case of FNAB).

### 4.3. Fluorescence-Activated Cell Sorting (FACS) Analysis

Cells obtained by FNAB and peripheral blood cells were subjected to extracellular labeling using a panel of fluorochrome-labeled antibodies specific for selected surface molecules. The concentration of used antibodies was standardized in accordance with the manufacturer’s instructions and adjusted to the number of cells in individual samples. The respective samples were subjected to multiple extracellular staining with following monoclonal antibodies (mAb):

- Fluorescein isothiocyanate (FITC) conjugated mAb: anti-CD16 (3G8, Mouse IgG1) (BD Biosciences Pharmingen, San Jose, CA, USA),
- Phycoerythrin (PE) conjugated mAb: anti-SLAN (M-DC8, Mouse IgG1) (Miltenyi Biotec, Bergisch Gladbach, Niemcy), anti-CD86 (2331 (FUN-1), Mouse IgG1) (BD Biosciences), anti-CD11c (B-ly6, Mouse IgG1) (BD Biosciences),
- Peridinin-chlorophyll-protein complex (PerCP) conjugated mAb: anti-CD14 (M5E2, Mouse IgG2a) (BD Biosciences), anti-CD19 (4G7, Mouse IgG1) (BD Biosciences),
- Allophycocyanin conjugated mAb: anti-CD3 (UCHT1, Mouse IgG1) (BD Biosciences), anti-BDCA1-(CD1c) (AD5-8E7, Mouse IgG2a) (Miltenyi Biotec), anti-BDCA2-(CD303) (AC144, Mouse IgG1) (Miltenyi Biotec), anti-BDCA3-(CD141) (AD5-14H12, Mouse IgG1) (Miltenyi Biotec),
- Phycoerythrin-cyanin conjugate 7 (PE-Cy7) conjugated mAb: anti-CD56 (B159, Mouse IgG1) (BD Biosciences).

Post-staining red blood cell lysis was performed using BD FACS lysing solution (BD Biosciences Pharmingen, San Jose, CA, USA). Before measurement samples were washed in PBS and centrifuged at 1400 RPM for 5 min at 20 °C. 

FACS measurements were performed with a BD FACSCanto II^®^ cytometer and BD FACSDiva^®^ software (BD Biosciences, San Jose, CA, USA). At least 10,000 cells were counted in mononuclear leukocyte gate in each sample.

**Figure 10:**
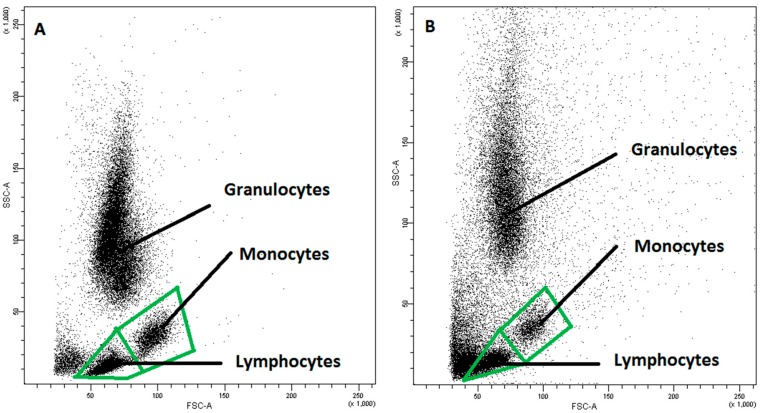
Exemplary plots of flow cytometry analysis showing the gating strategy of analyzed leukocyte populations in: (**A**) peripheral blood; (**B**) FNAB.

The population of mononuclear cells and its main fractions—lymphocytic and monocytic fractions were identified on the basis of size (FSC—forward side scatter) and granularity (SSC—side scatter) parameters (Figure 10).

**Figure 11:**
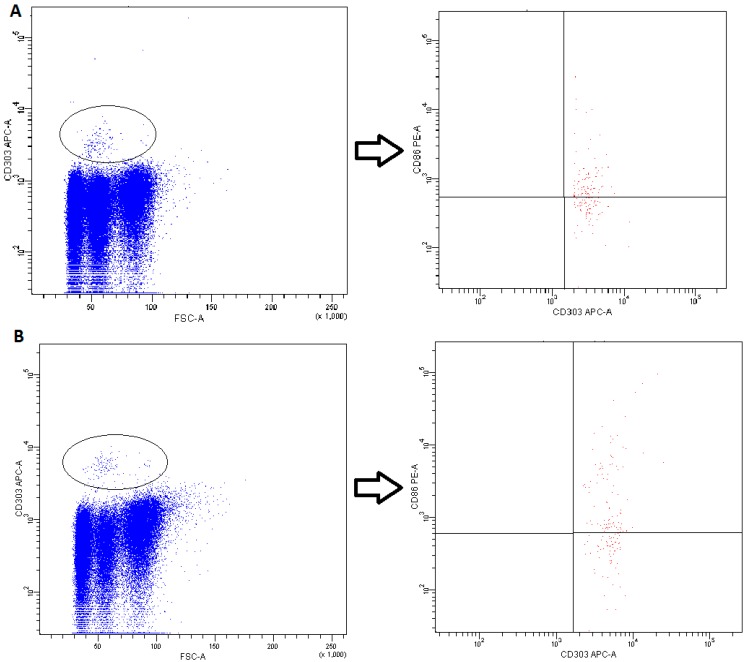
Exemplary plots of flow cytometry analysis showing the staining with antibodies specific for CD86 and CD303 in mononuclear leukocyte fraction: (**A**) peripheral blood; (**B**) FNAB. Gating of pDC (CD303^+^ cells) is presented.

**Figure 12:**
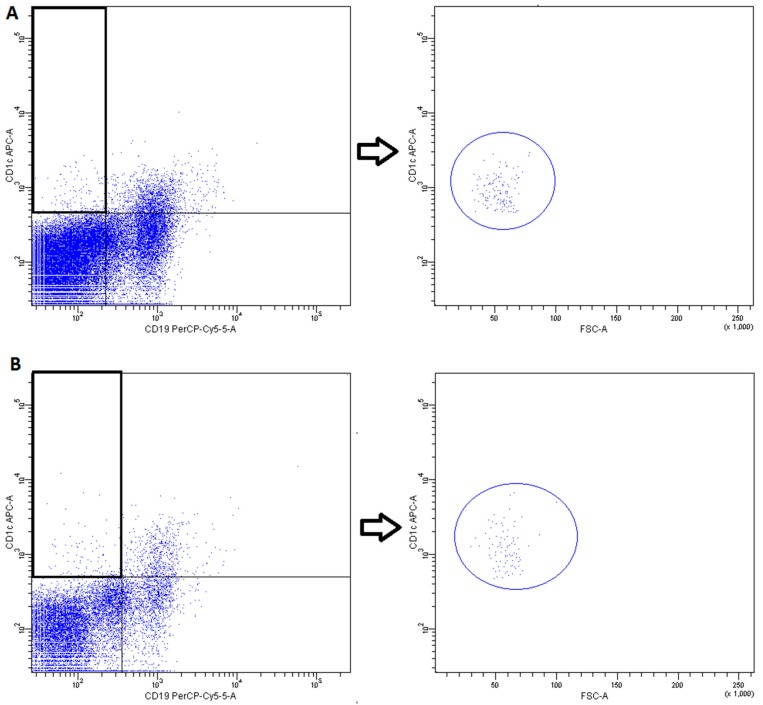
Exemplary plots of flow cytometry analysis showing the staining with antibodies specific for CD1c and CD19 in mononuclear leukocyte fraction: (**A**) peripheral blood (**B**) FNAB. Gating of cDC (CD1c^+^CD19^−^ cells) is presented.

**Figure 13:**
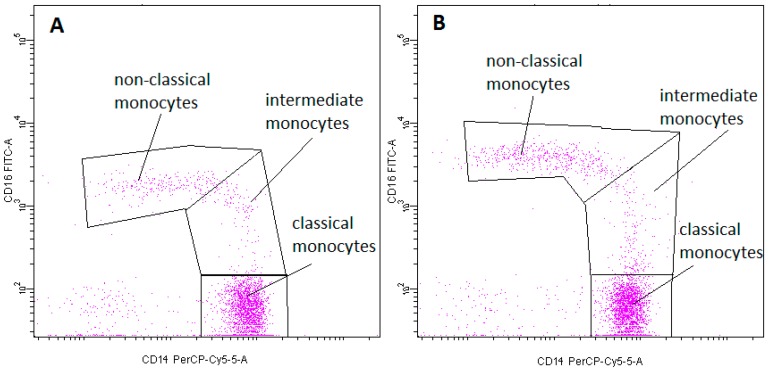
Exemplary plots of flow cytometry analysis showing the staining with antibodies specific for CD14 and CD16 in monocyte fraction: (**A**) peripheral blood (**B**) FNAB. Gating of individual monocyte subpopulations is presented: classic monocytes (CD14^high^CD16^−^), intermediate monocytes (CD14^high^CD16^+^), non-classical monocytes (CD14^low^CD16^+^).

**Figure 14:**
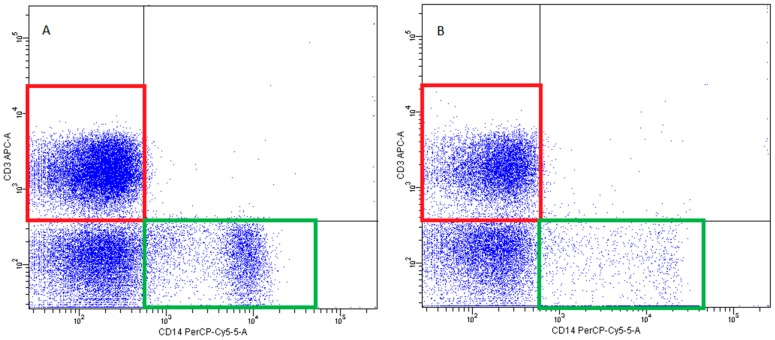
Exemplary plots of flow cytometry analysis showing the staining with antibodies specific for CD3 (T lymphocytes—red gates) and CD14 (monocytes—green gates) in mononuclear leukocyte fraction: (**A**) peripheral blood (**B**) FNAB.

**Figure 15:**
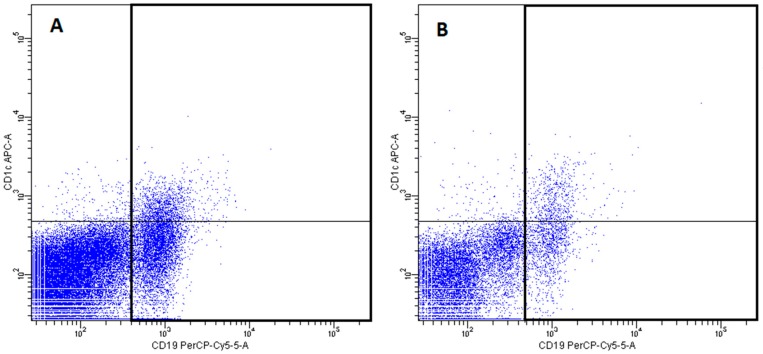
Exemplary plots of flow cytometry analysis showing the staining with antibodies specific for CD1c and CD19 in mononuclear leukocyte fraction: (**A**) peripheral blood (**B**) FNAB. Gating of B lymphocytes (CD19^+^ cells) is presented.

**Figure 16:**
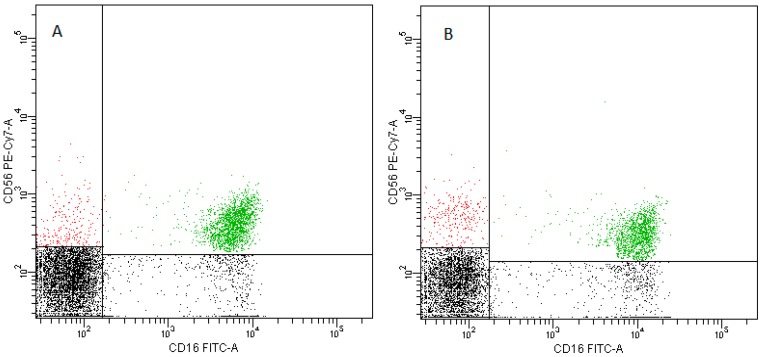
Exemplary plots of flow cytometry analysis showing the staining with antibodies specific for CD3 and CD56 in lymphocyte fraction: (**A**) peripheral blood (**B**) FNAB. Blue dots—CD3^−^CD56^+^ NK cells, red dots—CD3^+^CD56^+^ NKT cells.

**Figure 17:**
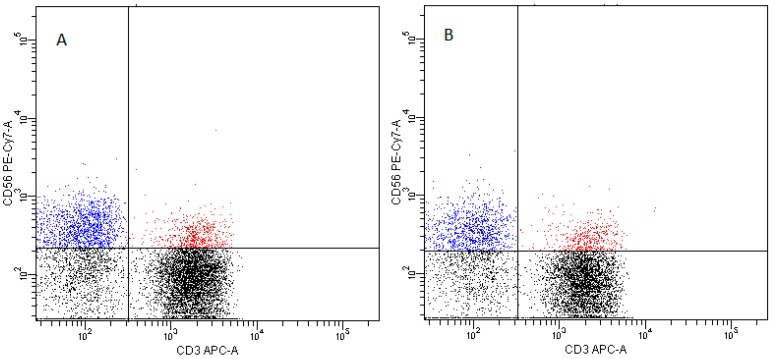
Exemplary plots of flow cytometry analysis showing the staining with antibodies specific for CD56 and CD16 in lymphocyte fraction: (**A**) peripheral blood B. FNAB. Red dots—CD3^−^CD56^+^CD16^−^ NK cells, green dots—CD3^−^CD56^+^CD16^+^ NK cells.

Individual populations of immune cells were identified by their expression of surface antigens:

- DCs subsets were recognized on the basis of expression of a panel of surface molecules known as Blood Dendritic Cell Antigens (BDCA) and CD19 [52].
  - pDCs—BDCA2 (CD303)^+^ (Figure 11)
  - cDCs—BDCA1 (CD1c)^+^ CD19^−^ (Figure 12)
- Monocyte subsets were recognized on the basis of the surface expression level of CD14 and CD16 molecules (Figure 13).
- T lymphocytes—CD3^+^ (Figure 14)
- B Lymphocyte—CD19^+^ (Figure 15)
- NK cells—CD3^−^CD56^+^ cells—subpopulations of NK cells were recognized on the basis of the surface expression level of CD16 molecule (Figure 16)
- NKT cells—CD3^+^CD56^+^ cells (Figure 17)

### 4.4. Statistical Analysis

The statistical analysis was carried out using the Statistica 12 software (Statsoft Polska, Kraków, Poland). A graphical representation of the results was prepared using the SigmaPlot 11 software (Systat Software Inc., San Jose, CA, USA). The normality of the distribution was assessed using the Shapiro–Wilk test. The Student’s *t*-test was used for normally distributed parameters. For matched pairs of parameters the Wilcoxon signed-rank test was used. In all the analyses, results were considered statistically significant when *p* < 0.05.

## 5. Conclusions

This study is one of the first attempts to gain insight into the structure of the regulatory cell population potentially involved in the immunopathological processes of thyroid focal lesions.

The results of parallel analysis in the peripheral blood and FNAB material suggest the existence of local, organ-specific immune reaction control networks, associated not only with the most effective APC—DCs and monocytes, but also with lymphocytic populations with known immunoregulatory properties such as NK and NKT cells.

In addition to the organ specificity, the differences found between individual groups of patients suggest a different involvement of the regulatory elements in the immunopathology of specific thyroid diseases. However, further research involving large clinically homogenous groups of patients is needed to elucidate potential differences between particular disorders of the thyroid, especially autoimmune conditions such as HT and GD.

These observations may be of potential value in further research aimed at the development of diagnostic and therapeutic tools.

## Figures and Tables

**Table 1:** Characteristics of the patients included in the study.

Parameters	All Patients				Non-AITD Patients				AITD Patients			
	Min.	Med.	Max.	Mean	Min.	Med.	Max.	Mean	Min.	Med.	Max.	Mean
Age (years)	23	52	85	53.37	23	53	85	54.02	31	52	85	52.57
TSH (µIU/mL)	0.01	1.15	67.3	2.85	0.01	1.04	67.3	3.34	0.01	1.47	6.96	2.05
fT3 (pg/mL)	1.12	3.12	9.5	3.20	1.22	3.11	5.3	3.22	2.58	3.1	4.14	3.17
fT4 (ng/dL)	0.85	1.28	10.07	1.46	0.86	1.25	10.07	1.47	0.89	1.31	1.78	1.33
anti-TPO (IU/mL)	<0.5	13.35	>600	59	<0.5	9.08	33	10.92	<0.5	104.87	>600	183.88
anti-Tg (IU/mL)	<10	20.8	484	85.37	<10	14.50	106.2	21.81	<10	31.51	471.7	139.72
anti-TSHR (IU/mL)	<0.3	0.32	7.44	0.61	<0.3	0.34	1.05	0.30	<0.3	0.3	7.44	0.77
Sex (F/M)	77/13				57/10				20/3			

Abbreviations: TSH, thyroid stimulating hormone, fT3, trijodothyronine; FT4, thyroxine; anti-TPO, thyroid peroxidase antybodies; anti-Tg, thyroglobulin antibodies; anti-TSHR, TSH receptor antibodies, F, female, M, male. Reference ranges: TSH 0.27–4.2 (µIU/mL); FT3 III 2.6–4.40 (pg/mL); FT4II 0.93–1.7 (ng/dL); anti-TG < 115 (IU/mL); anti-TPO < 34 (IU/mL); anti-TSHR < 1.75 (IU/mL). Min. (Minimum); Med. (Median); Max. (Maximum).

## Abbreviations

AITD	Autoimmune thyroid diseases
anti-Tg	Thyroglobulin antibodies
anti-TPO	Thyroid peroxidase antybodies
anti-TSHR	TSH receptor antibodies
APC	Antigen presenting cells
cDCs	Conventional DCs
DCs	Dendritic cells
FACS	Fluorescence-activated cell sorting
FcγRIII	Fc type G immunoglobin receptor
FITC	Fluorescein isothiocyanate
FNAB	Fine needle aspiration biopsy
FSC	Forward side scatter
fT3	Free triiodothyronine
fT4	Free thyroxine
GD	Graves’ disease
GM-CSF	Granulocyte/macrophage colony-stimulation factor
HT	Hashimoto’s thyroiditis
IFN	Interferon
Ig	Immunoglobulin
IL	Interleukin
LPS	Lipopolysaccharide
MHC	Major histocompatibility complex
NK	Natural killer (cells)
NKT	Natural killer T (cells)
PBMCs	Peripheral blood mononuclear cells
PBS	Phosphate buffered saline
pDCs	Plasmacytoid DCs
PE	Phycoerythrin
PerCP	Peridinin-chlorophyll-protein complex
PE-Cy7	Phycoerythrin-cyanin conjugate 7
PSGL-1	P-selectin glycoprotein ligand-1
slan	6-Sulfo LacNAc
SSC	Side scatter
Tg	Thyroglobulin
TGF-β	Transcription growth factor-β
Th	T helper (cell)
TNF	Tumor necrosis factor
TPO	Thyroid peroxidase
TR	Thyroid hormone receptor
TSH	Thyroid stimulating hormone
US	Ultrasonography
